# Autoimmunity: A Barrier to Gene Flow in Plants?

**DOI:** 10.1371/journal.pbio.0050262

**Published:** 2007-09-04

**Authors:** Liza Gross

Nearly 150 years after Darwin published *The Origin of Species*, evolutionary biologists are still working out the conditions and processes that give rise to new species. Central to the identity of a species—whether species exist in nature or as theoretical constructs, as some argue—is the ability to interbreed and produce fertile offspring. Several mechanisms can act as reproductive barriers, either before or after fertilization, to prevent gene flow between populations and set the stage for speciation.

One postfertilization mechanism in plants, called hybrid necrosis, arises from interactions between genes that cause misshapen, yellow, or damaged leaves and stunted growth, much like an infection. Hybrid necrosis occurs in crosses within and between species, suggesting that similar evolutionary processes may be at work at different times as the genes of the interbreeding species drift apart.

Conditions like hybrid necrosis, according to the classic Dobzhansky-Muller model of hybrid incompatibility, arise from deleterious interactions between genes inherited from the parents. As parental lineages diverge, the theory goes, each line evolves independent mutations that are harmless in the parent but prove detrimental when co-expressed in the hybrid. The evolutionary forces driving this divergence are not well understood, but adaptive evolution has been implicated in generating hybrid incompatibility, based on evidence that known incompatibility genes evolve rapidly.

In a new study, Kirsten Bomblies et al. investigate the genetic basis of hybrid necrosis in *Arabidopsis thaliana*, the favorite model organism of plant biologists. The researchers show that, in four cases of necrosis, the disorder results from the activation of genes normally involved in local responses to a pathogen attack, such as killing infected cells. In the hybrid, the genes interact to trigger a systemic immune response, leading to an autoimmunity-like condition characterized by cell death throughout the plant. Further evidence for this scenario comes from the identification of a common plant disease-resistance gene as a causative agent in one case of necrosis.

Bomblies et al. began investigating genetic incompatibilities when they noticed that hybrid progeny of two *A. thaliana* strains, Uk-1 and Uk-3, developed severe necrosis and failed to flower. Although all the offspring became necrotic at the mild temperature that *A. thaliana* encounters in the wild, they flowered at higher temperatures, allowing the researchers to collect seeds for genetic analysis.

**Figure:**
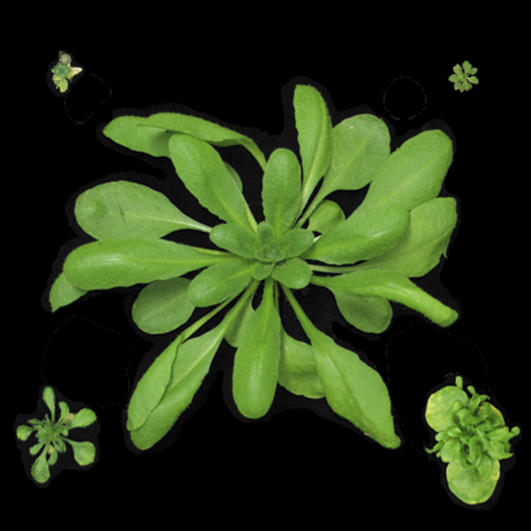
Incompatibilities between genes inherited from healthy parents (center) cause disorders like hybrid necrosis when combined in offspring, which develop yellowed misshapen leaves and stunted growth, and experience early death.

The researchers scanned the genomes of the afflicted plants for genetic variations that might contribute to necrosis and identified two regions, one inherited from each parent strain, located on different chromosomes. One locus (called *DM1*) was derived from the Uk-3 strain; the second, *DM2*, came from the Uk-1 plants. To isolate and analyze these potential incompatibility regions, the researchers first conducted a series of backcrosses, crossing the Uk-1/Uk-3 hybrids with a standard *A. thaliana* lab strain (Col-0) to transfer the unlinked loci to the same genomic background. They selected plants with clear signs of necrosis for subsequent crosses to ensure that they retained both loci. Over a few more backcrosses, and with the help of molecular markers, they generated Col-0 strains carrying either the Uk-1 region or the Uk-3 region and cloned each region for further analysis.

When the researchers separately inserted each region into the Col-0 strain, the resulting hybrid strains (Col-0/Uk-1 and Col-0/Uk-3) appeared normal. But when they crossed a Col-0 strain carrying the Uk-3 region with a Col-0/Uk-1 line, the offspring had the same necrotic symptoms as the Uk-1/Uk-3 hybrids. These experiments demonstrated not only that interactions between the Uk-3 region and the Uk-1 region cause necrosis but that a gene located within the Uk-3 region is all that is required for necrosis when the Uk-1 region is present.

The*DM1* region from the Uk-3 strain contains seven genes, including an *NB-LRR* gene; *NB-LRR* receptors are the most common type of disease-resistance genes in plants. (Over 150 have been identified in the *A. thaliana* genome.) *NB-LRR* mutations have previously been shown to cause lesions and retarded growth, consistent with inappropriate activation of plant immune responses and similar to necrosis in Uk-1/Uk-3 hybrids, implicating the *NB-LRR* gene as a candidate gene for *DM1*. By inhibiting the expression of several genes in the *DM1* region of one strain, the researchers confirmed that the *NB-LRR* gene was indeed the culprit. Only when *NB-LRR* was inactivated did hybrid plants appear normal, confirming the gene’s role in autoimmunity and necrosis. Supporting this conclusion, microarray analysis of leaves from sick plants showed a substantial increase in activity for genes involved in the immune response to infection, compared with normal plants. A similar bias was found in the gene expression profiles of other hybrids showing necrosis symptoms.

Having determined that the *NB-LRR* variant arose and persists in wild Arabidopsis plants, the researchers investigated the prevalence of hybrid necrosis among wild strains. About 2% of wild crosses developed the same necrotic symptoms as the Uk-1/Uk-3 hybrids, owing to five different sets of incompatible genes. And again, necrosis occurred at the environmentally relevant mild temperatures but not at higher temperatures.

Altogether, these results show how a genetic incompatibility syndrome arises when two genes that are normally harmless in a parental lineage turn out to have disastrous consequences for hybrid offspring. The researchers have not yet determined which gene in the *DM2* region of Uk-1 interacts with the *DM1* Uk-3 region, but they point out that *DM2* also contains *NB-LRR* genes. Among the possible scenarios this suggests is that selection pressures on disease-resistance genes engaged in host battles with pathogens caused the genes to diverge in the parental lineages to the point where they became incompatible in the hybrid genome. This link between hybrid necrosis and disease-resistance pathways has also been suggested in tobacco and tomato plants, indicating that this mechanism may act to erect postfertilization gene-flow barriers in diverse plant species. By establishing *A. thaliana* as a promising model for elucidating the agents of hybrid necrosis and other genetic incompatibilities, this study provides a framework for more fully exploring the role of disease-resistance genes in necrosis and the evolutionary forces that erect gene-flow barriers on the path to speciation.

